# Study on Reconstruction and Feature Tracking of Silicone Heart 3D Surface

**DOI:** 10.3390/s21227570

**Published:** 2021-11-14

**Authors:** Ziyan Zhang, Yan Liu, Jiawei Tian, Shan Liu, Bo Yang, Longhai Xiang, Lirong Yin, Wenfeng Zheng

**Affiliations:** 1 School of Innovation and Entrepreneurship, Xi’an Fanyi University, Xi’an 710105, China; ziyaan.zhang@outlook.com; 2 School of Automation, University of Electronic Science and Technology of China, Chengdu 610054, China; EEvian.Liu@gmail.com (Y.L.); jravis.tian23@gmail.com (J.T.); yangbo.sd@gmail.com (B.Y.); longhai.xiang@outlook.com (L.X.); wenfeng.zheng.cn@gmail.com (W.Z.); 3 Department of Geography and Anthropology, Louisiana State University, Baton Rouge, LA 70803, USA; yin.lyra@gmail.com

**Keywords:** Delaunay triangulation, reconstruction of three-dimensional surface, feature tracking, convolutional neural network

## Abstract

At present, feature-based 3D reconstruction and tracking technology is widely applied in the medical field. In minimally invasive surgery, the surgeon can achieve three-dimensional reconstruction through the images obtained by the endoscope in the human body, restore the three-dimensional scene of the area to be operated on, and track the motion of the soft tissue surface. This enables doctors to have a clearer understanding of the location depth of the surgical area, greatly reducing the negative impact of 2D image defects and ensuring smooth operation. In this study, firstly, the 3D coordinates of each feature point are calculated by using the parameters of the parallel binocular endoscope and the spatial geometric constraints. At the same time, the discrete feature points are divided into multiple triangles using the Delaunay triangulation method. Then, the 3D coordinates of feature points and the division results of each triangle are combined to complete the 3D surface reconstruction. Combined with the feature matching method based on convolutional neural network, feature tracking is realized by calculating the three-dimensional coordinate changes of the same feature point in different frames. Finally, experiments are carried out on the endoscope image to complete the 3D surface reconstruction and feature tracking.

## 1. Introduction

As an important branch in the field of computer vision, three-dimensional reconstruction has been applied to many image processing fields, such as computer animation, virtual reality and digital media creation [1], and image processing in minimally invasive surgery is also an important application field. In modern minimally invasive surgery, endoscopes are widely used to detect and treat diseases in various parts of the human body. This type of surgery is performed by introducing endoscopes and surgical equipment through natural holes or small incisions on the skin surface. Compared with traditional surgery, minimally invasive surgery greatly reduces surgical trauma and postoperative morbidity, and has a shorter recovery time. At the same time, minimally invasive surgery also has extremely high requirements for the surgeon’s position and direction perception during the operation, but the narrow field of observation during the operation makes it very difficult for the operator to sense the position of the endoscope and surgical equipment [2]. To overcome the inherent limitations of two-dimensional endoscopic images, computer-assisted surgery [3] is applied to surgical operations to provide doctors with accurate anatomical information in the patient’s body. Three-dimensional reconstruction provides doctors with a more intuitive three-dimensional model by processing the video sequence obtained by the stereoscopic endoscope. Such stereo perception can bring more qualitative and quantitative information about the shape of the tissue to the doctor. The tracking of feature points helps to describe the movement of the tissue surface, so it can be of great help to minimally invasive surgery or future automated robotic surgery.

Nowadays, feature-based 3D reconstruction and tracking technology is widely used in the medical field [4]. When performing minimally invasive surgery, the surgeon can realize three-dimensional reconstruction through the images obtained by the human body endoscope, restore the three-dimensional scene of the area to be operated, and track the movement of the soft tissue surface. This enables doctors to have a clearer understanding of the depth of the operation area, greatly reduces the negative impact of two-dimensional image defects, and ensures the smooth progress of the operation. However, the current application of feature-based 3D reconstruction technology in the medical field still has problems such as low matching accuracy of mid-feature points in endoscopic images and slow processing speed of image data. Therefore, the theory of feature-based three-dimensional reconstruction still has great research value, and it has great application value in medicine and other fields. The key to the research of feature-based 3D reconstruction and tracking technology lies in the processing of feature points in the image. The operation process of this kind of method can be roughly divided into three steps: feature detection, feature matching, 3D surface reconstruction and feature tracking.

3D surface reconstruction is the last step of feature-based 3D reconstruction [5]. Its main purpose is to build a more 3D visual object model based on images which has been considered an important information refinement in multiple fields [6,7]. The feature point cloud in 3D space can be determined by left and right image feature matching set, and then the 3D shape of the object can be restored by triangulation. In the case of large numbers of matching pairs of feature points, the dense reconstruction method can also be used [8,9,10,11].

The key to 3D surface reconstruction algorithms is to estimate the 3D shape of the object as accurately as possible, which is a process of mapping the original 2D image to 3D space by combining the feature point matching data [12,13,14]. Yang et al. [15] proposed a three-dimensional reconstruction method suitable for the case of less texture on the surface of the object. This method uses the depth information and color segmentation information in the image to achieve effective reconstruction of weak texture regions. To improve the real-time performance of the reconstruction method, Röhl et al. [16] proposed a method for intraoperative surface reconstruction, which optimizes the existing parallax correction, mesh generation and point cloud smoothing, and greatly improves the operation speed of the whole reconstruction process by using GPU. Ge et al. [17] proposed a 3D reconstruction method based on the orthogonal learning network. This method uses the feature point coordinates and the autocorrelation matrix extracted from the calibration data to train the orthogonal learning network. The minimum feature value of the training network is obtained, and the 3D coordinates are obtained through the corresponding relationship between the training network and the autocorrelation matrix, so as to obtain more robust and accurate reconstruction results. Song et al. [18] proposed a three-dimensional reconstruction method using synchronous positioning and composition technology. In this method, the real-time model of the object surface is gradually constructed by using the 3D deformation field which can recover the shape from the continuous image.

To improve the matching accuracy of feature points in the endoscope image and speed up image data processing, the following research and improvements are carried out:

1. In this study, firstly, the reconstruction method of 3D space points is studied, and then the 3D coordinates of feature points are recovered by combining camera parameters and geometric constraints.
2. Secondly, the reconstruction method of three-dimensional surface is studied. The surface is divided into several parts by Delaunay triangulation method. The surface reconstruction is realized by combining the restoration results of triangle area, and the feature tracking is realized by calculating the three-dimensional coordinate changes of feature points in different frames.
3. Finally, the experimental results of 3D surface reconstruction and feature tracking are analyzed.

## 2. Materials and Methods

### 2.1. Dataset

#### 2.1.1. Data Selection

The experiment in this section is carried out on the endoscope silica gel heart data obtained by the Da Vinci surgical robot, including 900 left and right video frames, with a frame rate of 25 frames per second. The rendering part of 3D surface is realized using OpenGL library.

#### 2.1.2. Data Processing

**Figure 1:**
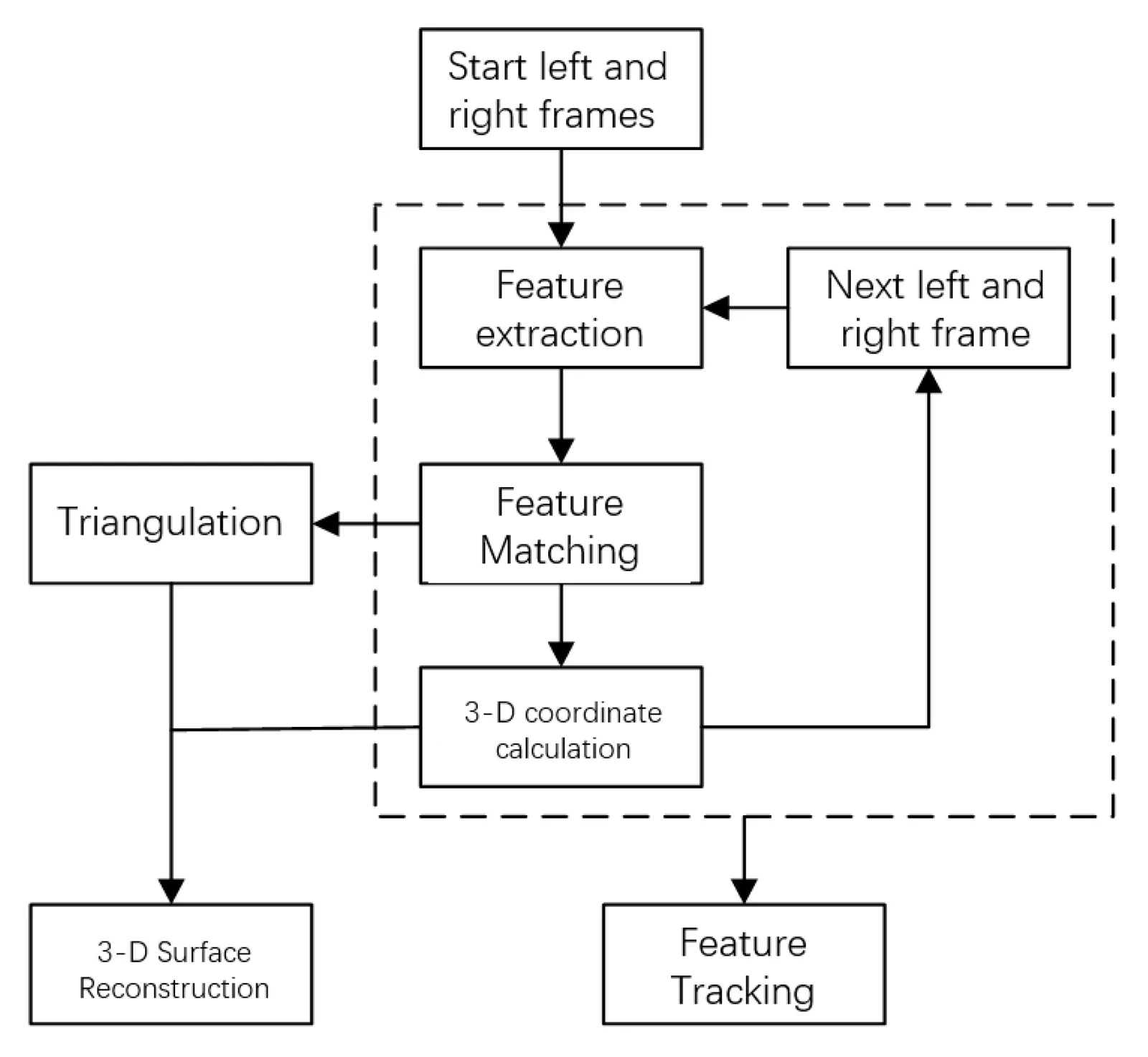
Flow chart of 3D surface reconstruction and feature tracking.

Three-dimensional surface reconstruction and feature tracking are the main contents of this paper. Its purpose is to use the feature matching information in two images to restore the visual three-dimensional shape of the object surface and track the feature points in the image [5]. Combining feature extraction method and feature matching method, 3D surface reconstruction is realized by matching feature points in the frame, calculating 3D coordinates of feature points, and triangulating discrete feature points on matching results; feature tracking is realized by calculating the position of the same feature point in different frames, and the calculation process is shown in Figure 1.

### 2.2. Feature Matching Method Based on Convolutional Neural Network

#### 2.2.1. Build the Framework of CNN

**Figure 2:**
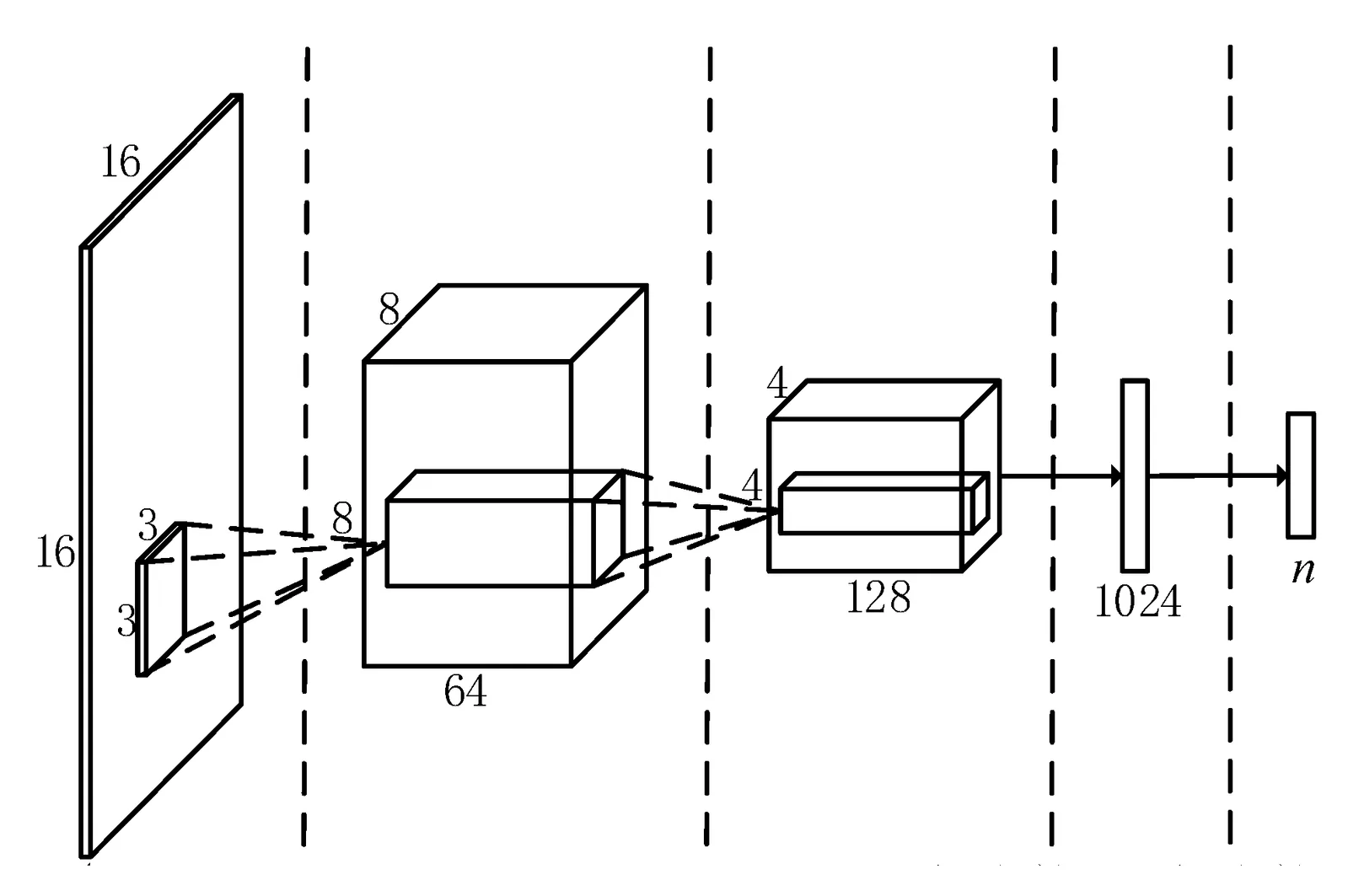
Overall architecture of convolutional neural network.

In this section, a network for the classification of endoscope video features is designed, and its overall architecture is shown in Figure 2. Taking into account the small amount of training data used in this topic, a simpler network structure is adopted to save network training time.

The entire convolutional neural network contains a total of 4 layers, the first two layers are convolutional layers, and the last two layers are fully connected layers. Among them, the first layer of convolution layer contains 64 convolution kernels, and the second layer contains 128 convolution kernels. The first layer of the fully connected layer contains 1024 nodes. To make the network adapt to different data training, the number of nodes in the second layer of fully connected layer corresponds to the number of feature point categories *n*. At the same time, the output of the last fully connected layer is sent to a Softmax transmission layer with *n* outputs to generate a probability distribution over *n* categories. The input image will be classified into the category with the highest probability, and the feature point classification is realized. To reduce network parameters and speed up training, each layer of convolutional layer is followed by a filter with a maximum pooling layer with a size of 2 × 2 pixels. To avoid gradient saturation during the network training process, all convolutional layers and fully connected layers use ReLU with relatively stable performance as the activation function. To reduce over-fitting, Dropout is introduced in the first fully connected layer.

#### 2.2.2. Feature Point Classification

The trained convolutional neural network can classify the image feature point blocks (16 × 16-pixel area centered on the feature) in the subsequent frames, so as to realize the classification of feature points. Mark the left and right images to be matched as $I_{l}$ and $I_{r}$ , respectively. First, the positions of the feature points in the two images still need to be determined. Use the improved FAST feature extraction algorithm in this paper to obtain the feature sets $F^{l}$ and $F^{r}$ in the two images, and use $f_{i}^{l}$ to represent the location coordinates of the i -th feature point in $F^{l}$ , and use $f_{i}^{r}$ to represent the location coordinates of the *i*-th feature point in $F^{r}$ . It should be noted that since traditional methods are not used for feature matching in the subsequent calculation process, there is no need to calculate the corresponding descriptors when extracting features here.

When judging the category of the feature point $f_{i}^{l}$ in the left picture, input its corresponding feature point block into the pre-trained convolutional neural network. Since the output of the network is the result of the distribution on multiple categories, the category of the feature point can be determined by the category with the highest probability. The final result obtained by the feature point is denoted as $o_{i}^{l}$ , as shown in Formula (1), where $c_{i}^{l}$ represents the category of the feature point, $P_{i}^{l}$ is the corresponding classification probability, and finally $O^{l}$ is used to represent all the feature points in the left picture after passing through the network The output collection. The output of the feature point block in the right figure after passing through the network is represented as $O_{i}^{r}$ in the same way, and $O^{r}$ is used to represent the set of all its classification results. In this way, after each image is judged by the convolutional neural network, all the feature points can get their corresponding categories and classification probabilities.

(1) $$o_{i}^{l}=\left[\begin{array}{cc} c_{i}^{l} & p_{i}^{l} \end{array}\right]$$

#### 2.2.3. Feature Matching Based on Classification Results

Feature points of the same category in different images can be regarded as matching feature points. However, when the feature classification results are used to achieve feature matching, since multiple feature points may belong to the same category in the classification results of all feature points in an image, the matching result cannot be directly obtained. Therefore, it is necessary to use the classification probability of each feature point to filter the feature points before matching, and only retain those feature points with stronger representative categories, so that at most one feature point of all categories in the same image is retained. The process of feature matching in this article can be divided into the following three steps:

(1) Build a matching matrix

To facilitate the calculation of the matching results, first establish matching matrices $M^{l}$ and $M^{r}$ to count the classification information of the feature points in the left and right images. Use $m_{i}^{l}$ ( i=1,2,…n ) to represent the i th element of $M^{l}$ (that is, the i th category), as shown in Equation (2), where $m_{i1}^{l}$ represents the position coordinate of the i th category in the left figure, and $m_{i2}^{l}$ represents that the point is the i th For the classification probability of each feature, all parameters in $m_{i1}^{l}$ and $m_{i2}^{l}$ are initialized to 0. $M^{r}$ and $M^{l}$ have the same structure and initialization method.

(2) $$m_{i}^{l}=\left[\begin{array}{cc} m_{i1}^{l} & m_{i2}^{l} \end{array}\right]$$

(2) Update matching matrix

Traverse the output $O^{l}$ of all the feature points in the left image. For $o_{i}^{l}$ , its category and classification probabilities are $c_{i}^{l}$ and $p_{i}^{l}$ , respectively. If the value of $p_{i}^{l}$ is greater than $m_{c_{i}^{l}2}^{l}$ , that is, the current feature point is more representative in category $c_{i}^{l}$ than the feature point stored in the matching matrix, then the matching matrix is updated, otherwise no operation is performed. The update process of the matching matrix is shown in Formula (3).

(3) $$\{\begin{array}{l} m_{i1}^{l}=f_{i}^{l}\\ m_{i2}^{l}=p_{i}^{l} \end{array}$$

Use $O^{r}$ to update $M^{r}$ in the same way. The update process of the matching matrix is the process of filtering the feature points. The feature points belonging to the same category in the same image will only retain the feature points with the highest classification probability after the update is completed.

(3) Calculate the matching result

Use matrix M to represent the final matching result, retrieve all elements in the two matching matrices, if $m_{i2}^{l}$ and $m_{i2}^{r}$ ( i=1,2,…n ) are not 0 at the same time, that is, the feature points of category F can be found in the left and right images, then $\left[\begin{array}{cc} m_{i1}^{l} & m_{i1}^{r} \end{array}\right]$ is added to in M , otherwise no operation is performed. Finally, output v as the feature matching result of $I_{l}$ and $I_{r}$ .

### 2.3. 3D Point Coordinate Recovery

To map the pixels in the image to the 3D space, we need not only the coordinates of the point in at least two images with different viewing angles, but also the geometric relationship between the camera and the pixels in the space [6]. The position of the same pixel on different imaging surfaces can be obtained by feature matching. This section will introduce the knowledge of epipolar geometry, and further study the restoration method of 3D coordinates of binocular camera pixels.

#### 2.3.1. Polar Geometry

**Figure 3:**
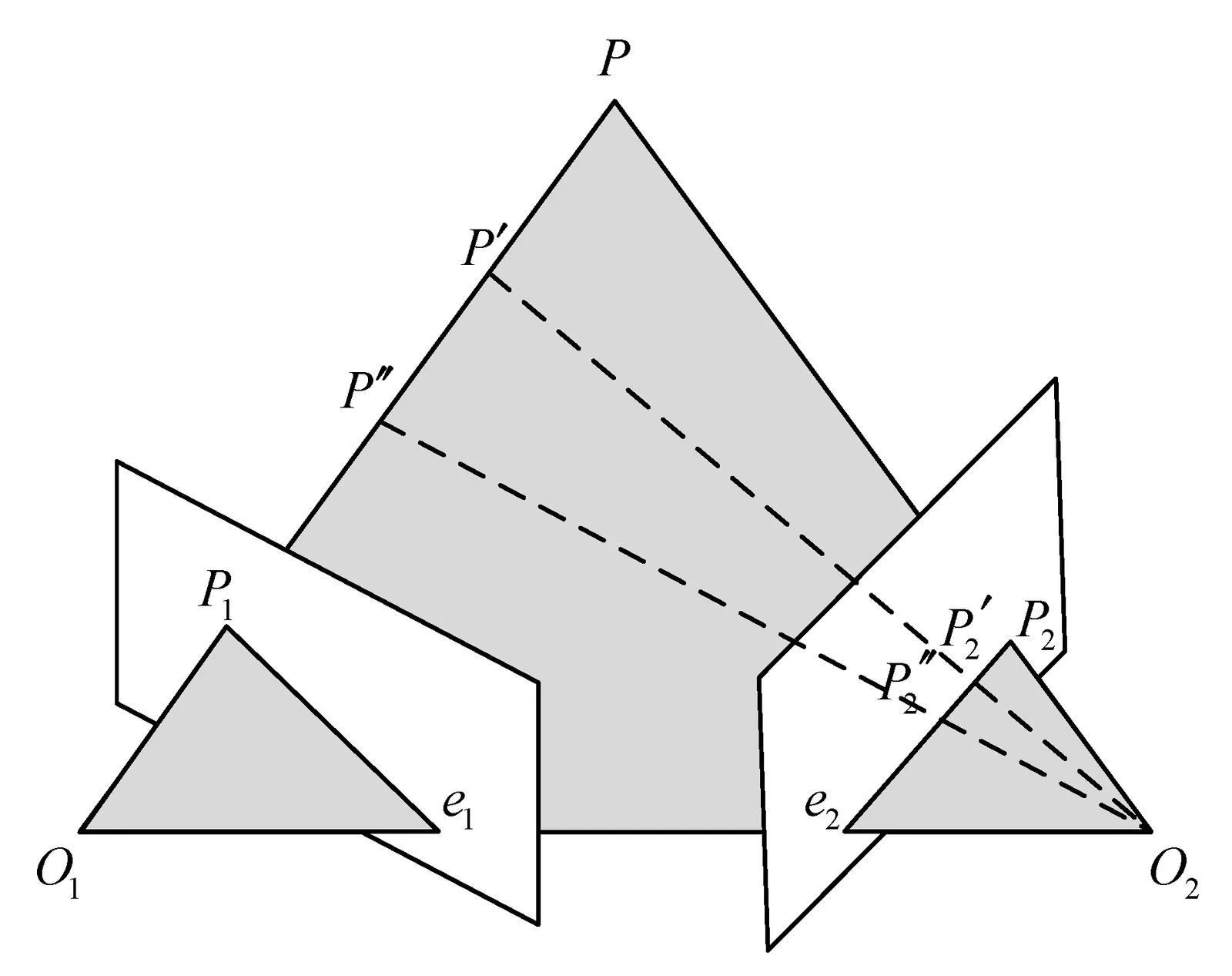
Polar geometry relationship.

Polar geometry is used to describe the geometric relationship between the images of the same object in three-dimensional space from different perspectives at the same time. The relationship is only related to the parameters of the camera and the relative attitude of the two images. A typical binocular imaging system is shown in Figure 3, where $O_{1}$ and $O_{2}$ represent the origin of the coordinate system of the left and right cameras, P is a point in three-dimensional space, and $P_{1}$ and $P_{2}$ are the positions of P on the imaging plane of the two cameras. The distance between the origin of the two camera coordinate systems, that is, the line between $O_{1}$ and $O_{2}$ , is called the baseline. The intersection of the two imaging planes and the baseline is called several points, which are represented by $e_{1}$ and $e_{2}$ , respectively.

The connection between $P_{1}$ and $e_{1}$ , $P_{2}$ and $e_{2}$ is called the polar line. Such a spatial relationship has an important property: P , $O_{1}$ , $O_{2}$ , $e_{1}$ , $e_{2}$ are coplanar, which is also called the polar plane. When the shooting position of the two cameras remains unchanged and point P moves to $P^{\prime }$ and $P^{\prime \prime }$ on the line between P and $O_{1}$ , the imaging point position of point P on the left imaging plane is still $P_{1}$ , which will not change; since the projection point of P on the right imaging plane is also on the polar plane, the imaging position of P on the right imaging plane must be on the polar line of the right plane.

This geometric relationship has a very important application in the feature matching process of left and right images of endoscope. When the position of the two endoscopes is known, for a detected feature point in the left image, when searching for a matching point in the right image, according to the geometric relationship of the opposite pole, it is only necessary to search near the polar line of the right plane (considering that there may be some errors). However, this method requires more camera parameters and requires two cameras to shoot at fixed positions, which requires high stability of the experimental environment in practical application.

#### 2.3.2. Geometric Relationship of the Binocular Camera

**Figure 4:**
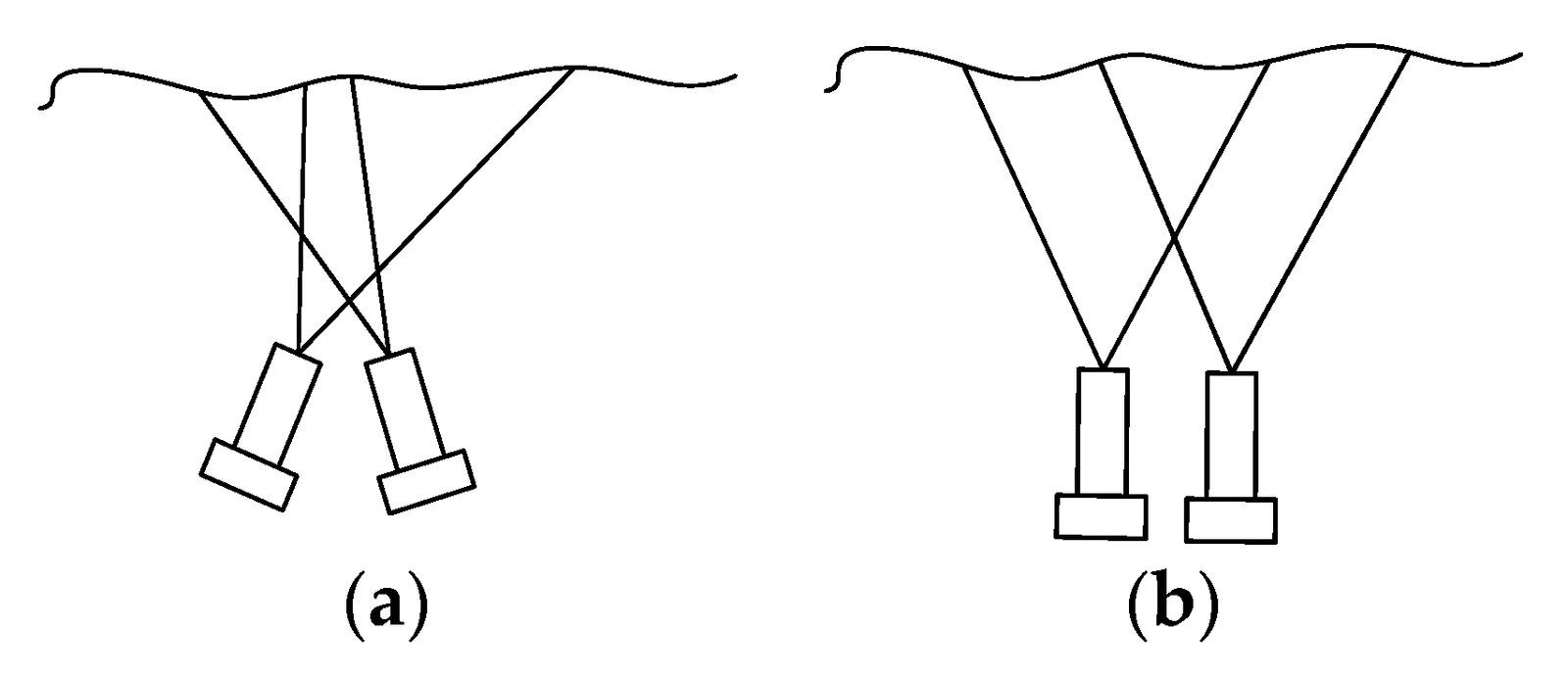
Placement of binocular camera. (**a**) Convergent; (**b**) parallel.

The endoscope used in minimally invasive surgery can be divided into monocular camera and binocular camera, in which binocular camera is to obtain the image in the scene by placing two cameras at different positions at the same time, so that the coordinates of a point in the two images can be recovered in the three-dimensional space through the corresponding coordinates and triangular geometric relationship [10]. According to the position relationship between the two cameras, the endoscope can be divided into convergent type and parallel type. The two cameras of the converging binocular camera are placed at a certain angle, while the two cameras of the parallel binocular camera are placed in parallel, as shown in Figure 4. The imaging models of cameras placed in different ways are also different.

**Figure 5:**
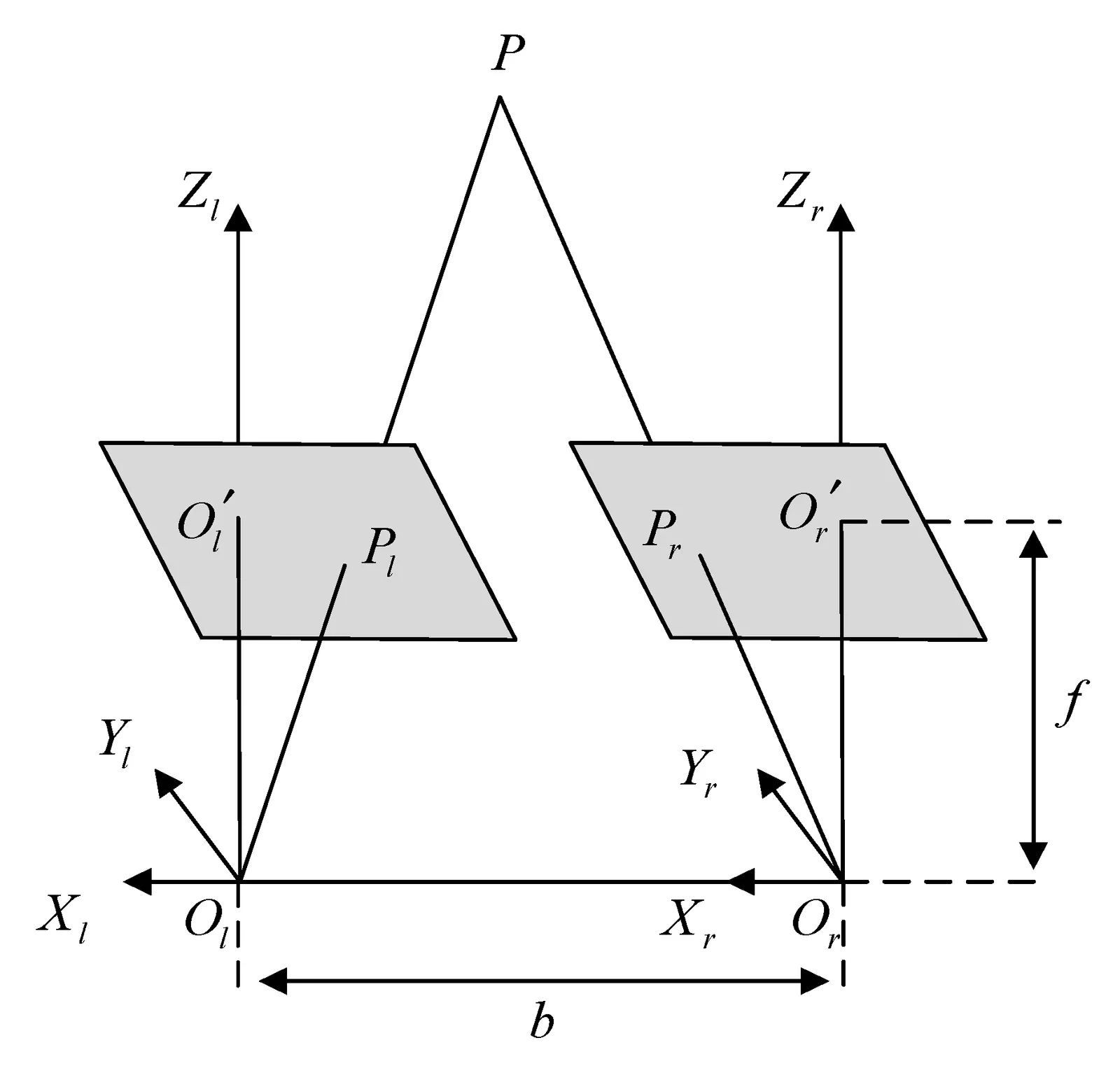
Geometric relationship of parallel binocular camera imaging.

Because of its simple structure and easy calculation, the parallel binocular camera is widely used in the field of minimally invasive surgery. When two cameras are placed in parallel, the line between the optical centers of the two cameras is parallel to the two imaging planes, and the two optical axes are perpendicular to the imaging plane. Thus, only horizontal parallax exists in the corresponding points of the images acquired by the two cameras. When two cameras have the same focal length, the imaging geometric relationship is shown in Figure 5.

$O_{l}$ and $O_{r}$ in Figure 5 represent the optical centers of left and right cameras respectively. The length of $O_{l}$ and $O_{r}$ lines is b , and the focal length of both cameras is f . The coordinates of a point P in three-dimensional space on two camera imaging planes are represented by $P_{l}=\left(x_{l},y_{l}\right)$ and $P_{r}=\left(x_{r},y_{r}\right)$ , respectively, and the coordinate of P in the left camera coordinate system is (X,Y,Z) . Furthermore, since the imaging point has no parallax in the $Y_{l}$ -axial direction, $y=y_{l}=y_{r}$ can be obtained by the similar triangle principle (4).

(4) $$\{\begin{array}{c} x_{l}=f\frac{X}{Z}\\ x_{r}=f\frac{X-b}{Z}\\ y=f\frac{Y}{Z} \end{array}$$

Let the horizontal parallax $d=x_{l}-x_{r}$ of point P in the two images; combined with (4), we can obtain the three-dimensional coordinates of corresponding points in the left and right images in space, as shown in (5). Therefore, for the matching feature points in the left and right images of the same frame in the parallel binocular endoscope, the three-dimensional coordinates in the left camera coordinate system can be obtained by combining (5) and binocular geometric constraints.

(5) $$\{\begin{array}{l} X=\frac{bx_{l}}{d}\\ Y=\frac{by}{d}\\ Z=\frac{bf}{d} \end{array}$$

### 2.4. Triangulation

After obtaining the 3D coordinate information of all the feature points in the endoscope image, the shape of the soft tissue surface in the image needs to be materialized. However, because the feature data obtained are discrete points in space, it is impossible to reconstruct the soft tissue directly. Through triangulation, the surface of soft tissue can be divided into several non-overlapping triangular areas, and then the multiple triangular areas are mapped to three-dimensional space, and then stitched together to form a more vivid surface shape.

#### 2.4.1. Delaunay Triangulation

**Figure 6:**
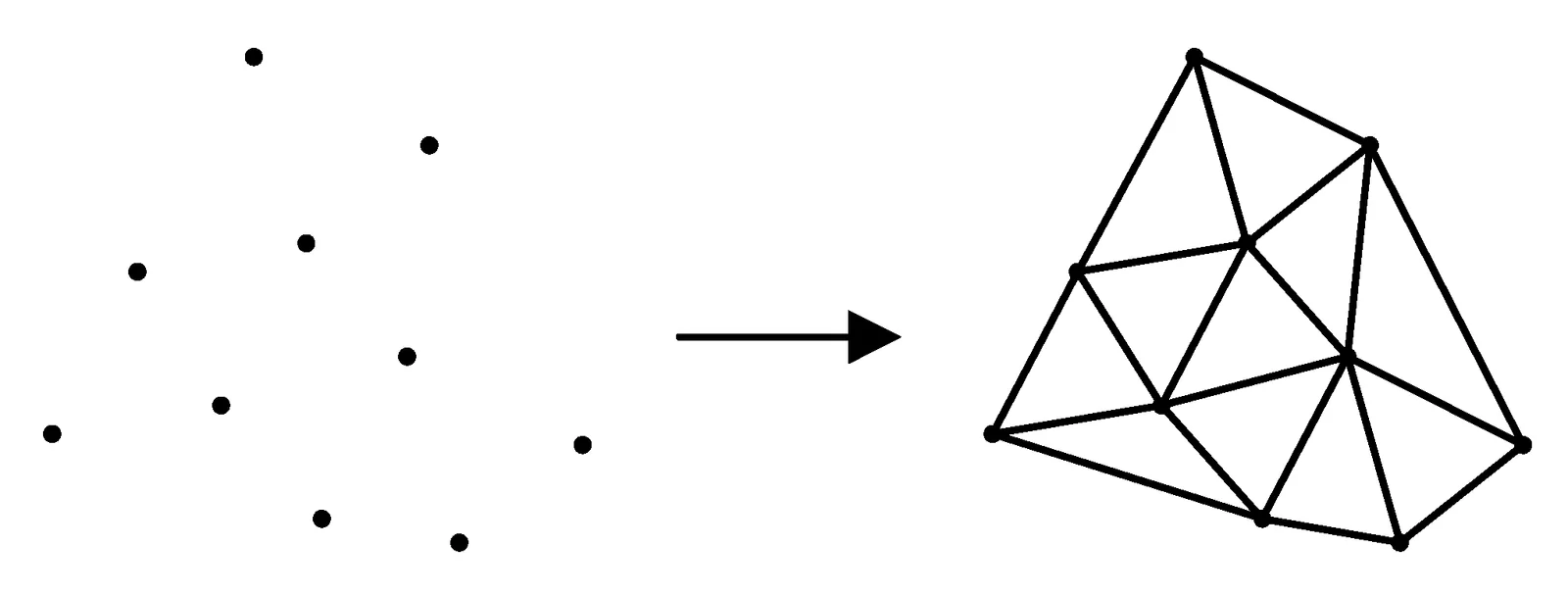
Delaunay triangulation.

Triangulation is the division of several discrete points in a plane into multiple disjointed triangular surfaces by means of lines between points. Any two triangles among the total number of triangles have at most one common edge. Delaunay triangulation is a special triangulation method. The triangulation results obtained by this method have many characteristics, among which the most important are the characteristics of the empty circle and the maximum minimum angle. The Delaunay triangulation results of discrete points in two-dimensional plane are shown in Figure 6.

The empty circle feature of Delaunay triangulation refers to the fact that the circumcircle of all triangles after triangulation does not contain other points; the maximum minimum angle feature refers to the fact that the minimum angle of all triangles after triangulation is the largest among all the triangulation methods of these points.

In addition, Delaunay triangulation has many excellent characteristics:

Nearest: A triangle is formed by the three nearest neighbors, and the line segments (the sides of the triangle) do not intersect.

Uniqueness: No matter where the area is constructed, there is only one final result (provided that there is no four-point circle).

Optimality: If the diagonals of the convex quadrilateral formed by any two adjacent triangles can be interchanged, then the smallest angle among the six internal angles of the two triangles will not become larger.

The most regular: If the smallest angle of each triangle in the triangulation is arranged in ascending order, the Delaunay triangulation will obtain the highest value.

Regional: adding, deleting, or moving a vertex will only affect the adjacent triangle.

Convex polygonal shell: The outermost boundary of the triangular mesh forms a convex polygonal shell.

In theory, in order to construct the Delaunay triangulation, Lawson proposed the local optimization process LOP (Local Optimization Procedure). Generally, the triangulation can be guaranteed to become a Delaunay triangulation after LOP processing. The basic method is as follows:

(1) Combine two triangles with common sides into a polygon.
(2) Check with the maximum empty circle criterion to see if the fourth vertex is within the circumcircle of the triangle.
(3) If it is, the correction diagonal is about to reverse the diagonal, that is, the processing of the local optimization process is completed.

**Figure 7:**
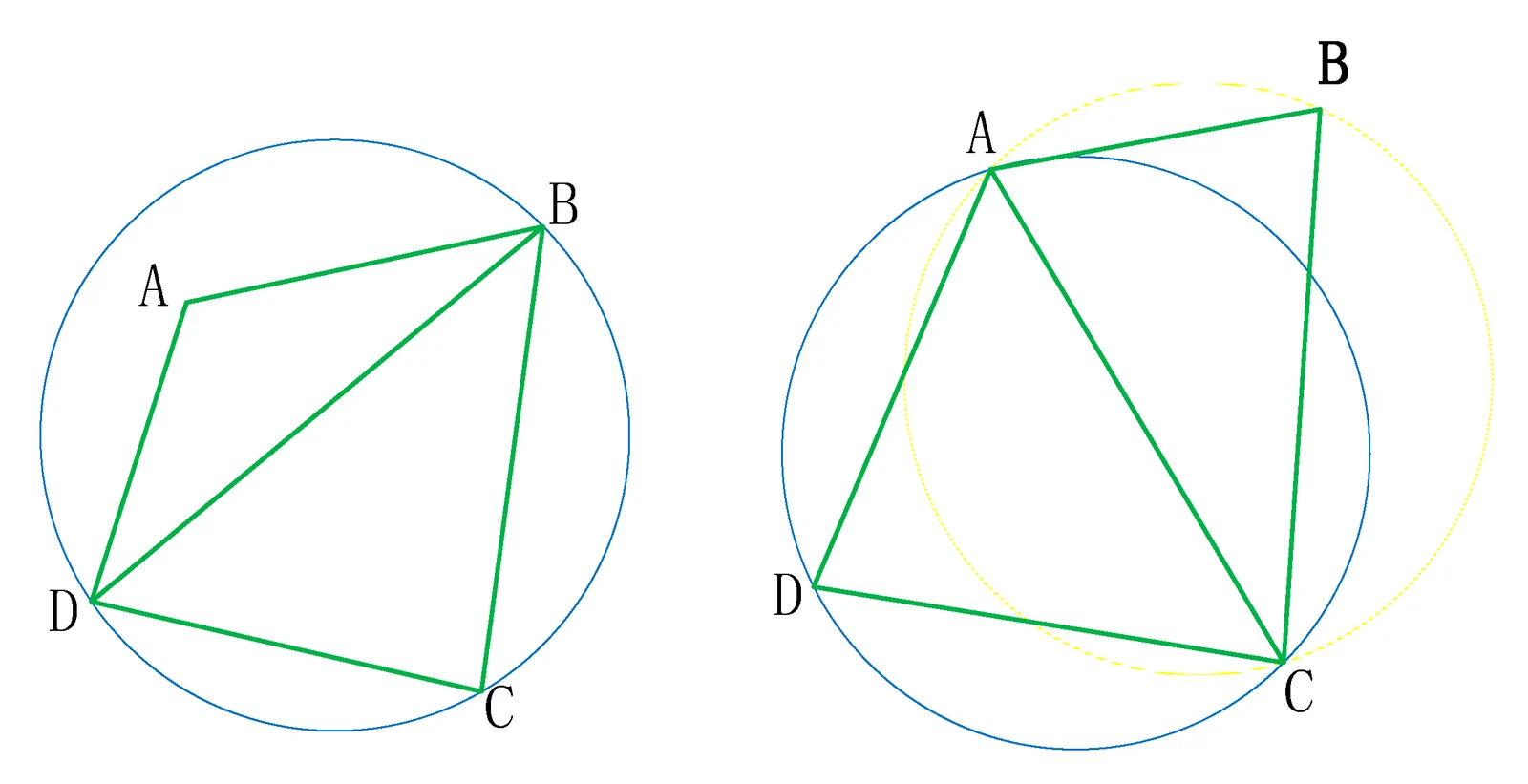
The LOP processing process.

The LOP processing process is shown in Figure 7. The circumscribed circle of △BCD on the left of the figure below contains point A, then execute (3), after execution, the right of the figure below.

Because Delaunay triangulation has many characteristics, this study uses this method to realize the triangulation of discrete feature points, so as to obtain a more vivid surface.

There are many methods to realize Delaunay triangulation, which can be divided into the divide and conquer algorithm, the point-by-point insertion method and the triangulation generation method. Among them, triangulation is the most time-consuming method, and its low efficiency has made it less widely used. The divide and conquer algorithm is less time-consuming, but the memory consumption of the algorithm is high. The point-by-point insertion method takes less space and has higher time efficiency, and the algorithm is simple.

To ensure less computing time and less running memory, the point-by-point insertion method is used to triangulate feature points. In 1977, Lawson proposed the algorithm for performing Delaunay triangulation using the point-by-point insertion method. After that, Lee and Schachlter, Bowyer, Watson, and Sloan successively developed and improved on this. Their algorithms have their own characteristics in the establishment of the initial triangulation method, the process of positioning the triangle where the point is located, and the process of insertion. This article illustrates two point-by-point insertion algorithms: the Lawson algorithm and the Bowyer–Watson algorithm.

(1) Lawson algorithm

The Lawson algorithm for point-by-point insertion was proposed by Lawson in 1977. The algorithm has a simple idea and is easy to program. The basic principle is: first create a large triangle or polygon, enclose all the data points, and insert a point into it. This point is connected with the three vertices of the triangle containing it to form three new triangles; then, pair them one by one. Carry out the empty circumscribed circle detection; at the same time, use the local optimization process (LOP) designed by Lawson for optimization, that is, through the method of swapping the diagonals to ensure that the formed triangulation is a Delaunay triangulation.

Advantages: The above-mentioned scatter-based network construction algorithm has rigorous theory and good uniqueness, and the grid satisfies the characteristics of empty circles, which is ideal. From the point-by-point insertion process, it can be seen that when a non-Delaunay edge is encountered, a new Delaunay edge can be constructed and formed by deleting and adjusting it. After completing the network construction, when adding new points, there is no need to re-network all the points, only the local network of the triangle affected by the new points, and the local network method is simple and easy to implement. Similarly, the deletion and movement of points can also be carried out quickly and dynamically.

**Figure 8:**
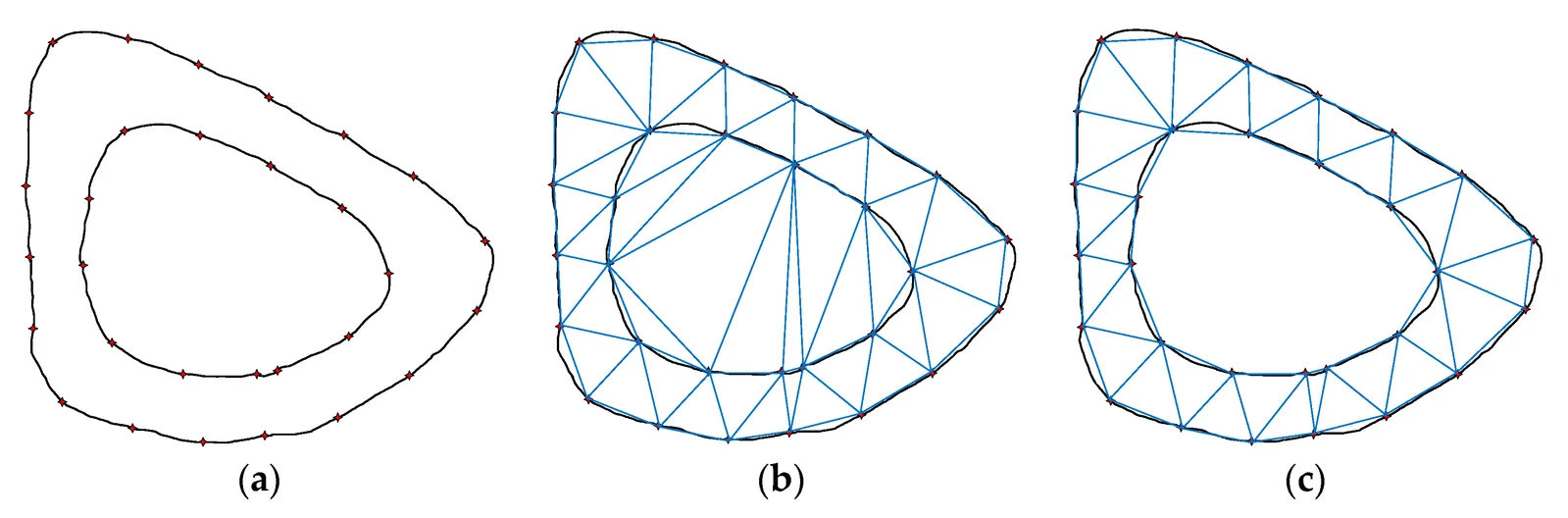
Example of Lawson algorithm: (**a**) original image; (**b**) error graph; (**c**) correct figure.

Disadvantages: However, in practical applications, this network construction algorithm is slower when the point set is large. If the point set range is a non-convex area or there is an inner ring, an illegal triangle will be generated. As shown in Figure 8, when a set of discrete points forms a circle, an illegal triangle is generated by the Lawson algorithm.

(2) Bowyer–Watson algorithm

The basic steps of the Bowyer–Watson algorithm for point-by-point insertion are:

1 Construct a super triangle, including all the scattered points, and put it into the triangle linked list.

2 Insert the scattered points in the point concentration one by one, find the triangle whose circumcircle contains the insertion point in the triangle linked list (called the influence triangle of the point), delete the common side of the influence triangle, and set the insertion point with all the vertices of the influence triangle Connect to complete the insertion of a point in the Delaunay triangle linked list.

3 According to the optimization criteria, optimize the newly formed triangles locally. Put the formed triangle into the Delaunay triangle list.

4 Execute step 2 in a loop until all the scattered points are inserted.

**Figure 9:**
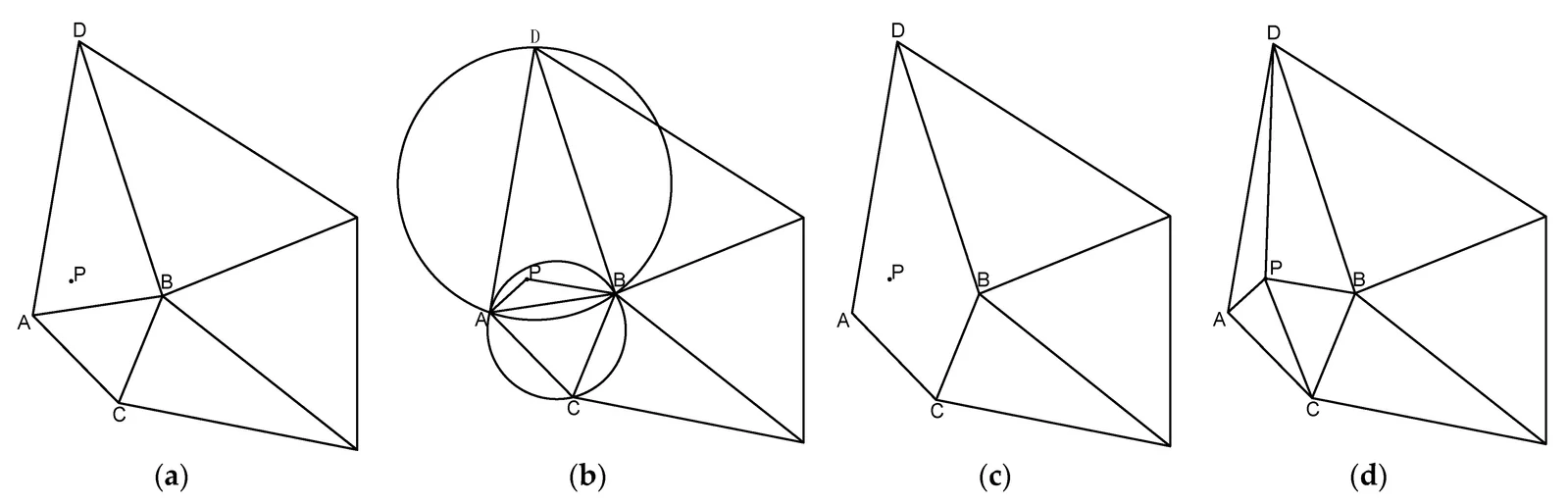
The key step 2 of the Bowyer–Watson algorithm. (**a**) Insert new node P; (**b**) decide how to connect P with other vertices; (**c**) delete edge AB; (**d**) form a triangle.

The key step 2 of this algorithm is illustrated in Figure 9:

#### 2.4.2. Triangulation of 3D Curved Surface

The discrete points of a three-dimensional curved surface can also be triangulated. Due to the increase in the dimensions of the coordinate points, the subdivision method is more complex. The current curved surface subdivision methods can be roughly divided into direct subdivision methods and plane projection methods.

(1) Direct dissection

As the name implies, the direct dissection method directly uses the three-dimensional point coordinates for subdivision. The first method does not change the topological structure of the origin set V , and uses a method similar to two-dimensional plane triangulation. V is continuously inserted, but the constraints used are different from plane triangulation, and its computational complexity is also higher; the second kind of method is to use triangulation to approximate the surface under the condition of allowing a certain amount of error, and the position and number of vertices in the results after subdivision will be different from the origin set V , so it is not suitable for occasions where the accuracy of the original data should be higher. Due to the large computation requirements, there is less research on the algorithm of direct subdivision.

(2) Plane projection method

The plane projection method requires less computation. The main idea of this method is to project the set of points V in 3D space onto a 2D plane to obtain the set of projection points $V^{\prime }$ ; then, $V^{\prime }$ is triangulated using a plane subdivision algorithm, and then the connection relationship between the points in the $V^{\prime }$ triangulation is mapped to V , so as to realize the triangulation of the surface in 3D space. The essence of the plane projection method is triangulation of plane point set. Because of its easy-to-understand calculation process and high calculation efficiency, and the fact that the subdivision results are applicable in many situations, it is more widely used.

Considering that the image used in this study is an intraoperative image collected by endoscope, less occlusion and overlap are present in the image. Considering the large number of calculations required for the direct subdivision method, this paper adopts the plane projection method with higher efficiency to triangulate the discrete feature points.

### 2.5. Feature Point Tracking

**Figure 10:**
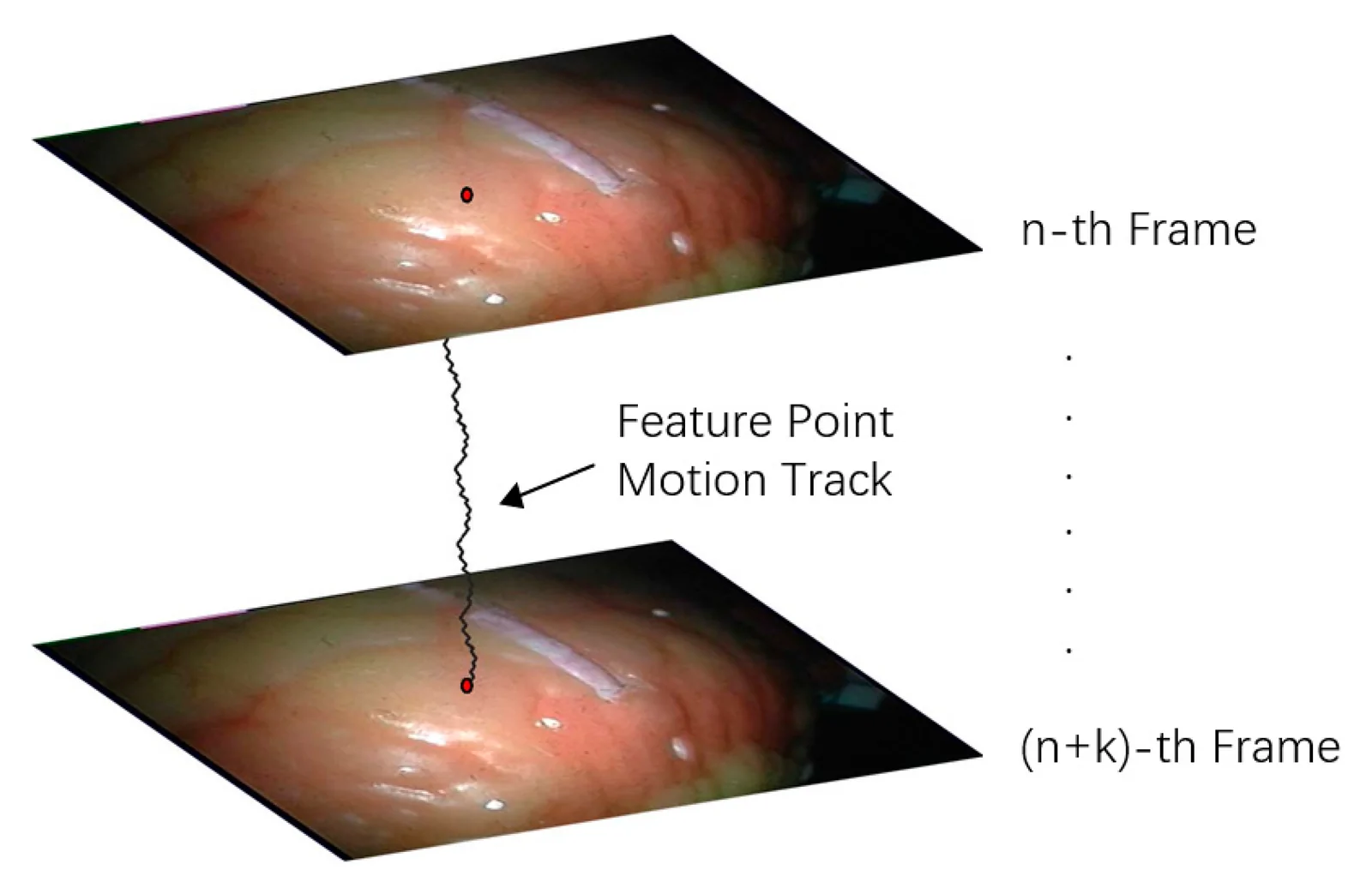
Feature point tracking diagram.

Feature point tracking refers to tracking the 3D spatial coordinates of pixels in a certain length of video sequence. Tracking the position changes of multiple points is conducive to the shape change analysis of the target area. The tracking of feature points in endoscopic video is shown in Figure 10.

To realize the 3D motion tracking of feature points in different frames, it is necessary to calculate the position of the tracking feature points in different frames. In the model-based 3D reconstruction method, since the tracking interest point area is extracted in each frame for calculation, the position tracking of feature points can be obtained using a deformation model [19]. In feature-based 3D motion tracking, feature point tracking methods can be roughly divided into two types.

The first method is to select a frame with better performance as the sample frame in the video sequence. When tracking the feature points in the sample, the subsequent frame and the sample frame are matched to obtain the change of the feature points. However, this method uses fixed feature matching samples in the process of feature tracking. Because the shape of feature points may change greatly in subsequent video frames, the tracking may be inaccurate, or could even fail. The other method does not select a specific sample frame, but selects the previous frame as the sample frame in each match, and dynamically updates the position and shape information of the tracked feature points. This tracking method takes into account the possible morphological changes of feature points. However, due to the use of the adjacent frame tracking strategy, if there is an error or tracking failure in the tracking process, the follow-up feature tracking results will be seriously affected, and may even be unable to recover the tracking.

By constructing a decision tree to classify feature points or extended Kalman filter, the above defects can be solved to a certain extent and feature point tracking can be realized [20], but the tracking effect is still not ideal. In this paper, a convolutional neural network is used to classify the feature points. The characteristics of all the feature points are preserved in the network training stage, which has higher robustness to the changing feature points. Therefore, different strategies can be used to track the feature points.

Because this study realizes feature matching by constructing a convolutional neural network, it does not need to calculate the descriptors of each frame when tracking the feature points, which avoids the defects caused by the insufficient performance of the descriptors. The feature points of the tracked category are found in each frame of the endoscope video [21], and the 3D coordinates are recovered by the binocular geometric relationship to complete the tracking. In this paper, the tracking process of feature points can be realized by iterative calculation.

(1) For the feature points to be tracked in the initial frame, the corresponding category in convolutional neural network is found, and the feature points of the category are found in the left and right frames of the initial tracking. The three-dimensional coordinates of the feature points are calculated as the initial tracking positions by binocular geometric constraints.

(2) Find the same type of feature points in the next left and right frames, and calculate their 3D coordinates. If the corresponding classification feature points cannot be found in any one of the left and right images, the coordinates of the feature points of the frame will be marked as blank, indicating that the frame tracking has failed.

(3) If the number of iterations reaches the desired tracking frame, stop; otherwise return to step (2).

Through the above methods, we can determine the 3D coordinate changes of a certain class of feature points on the continuous video sequence, and then realize the feature tracking. Because the coordinate calculation of feature points in each frame is calculated by the classification results of convolutional neural network, even if the tracking failure of feature points or the error of matching results in a certain frame, the calculation of feature point coordinates in subsequent frames will not be affected.

## 3. Results

### 3.1. 3D Curved Surface Reconstruction Experiment

**Figure 11:**
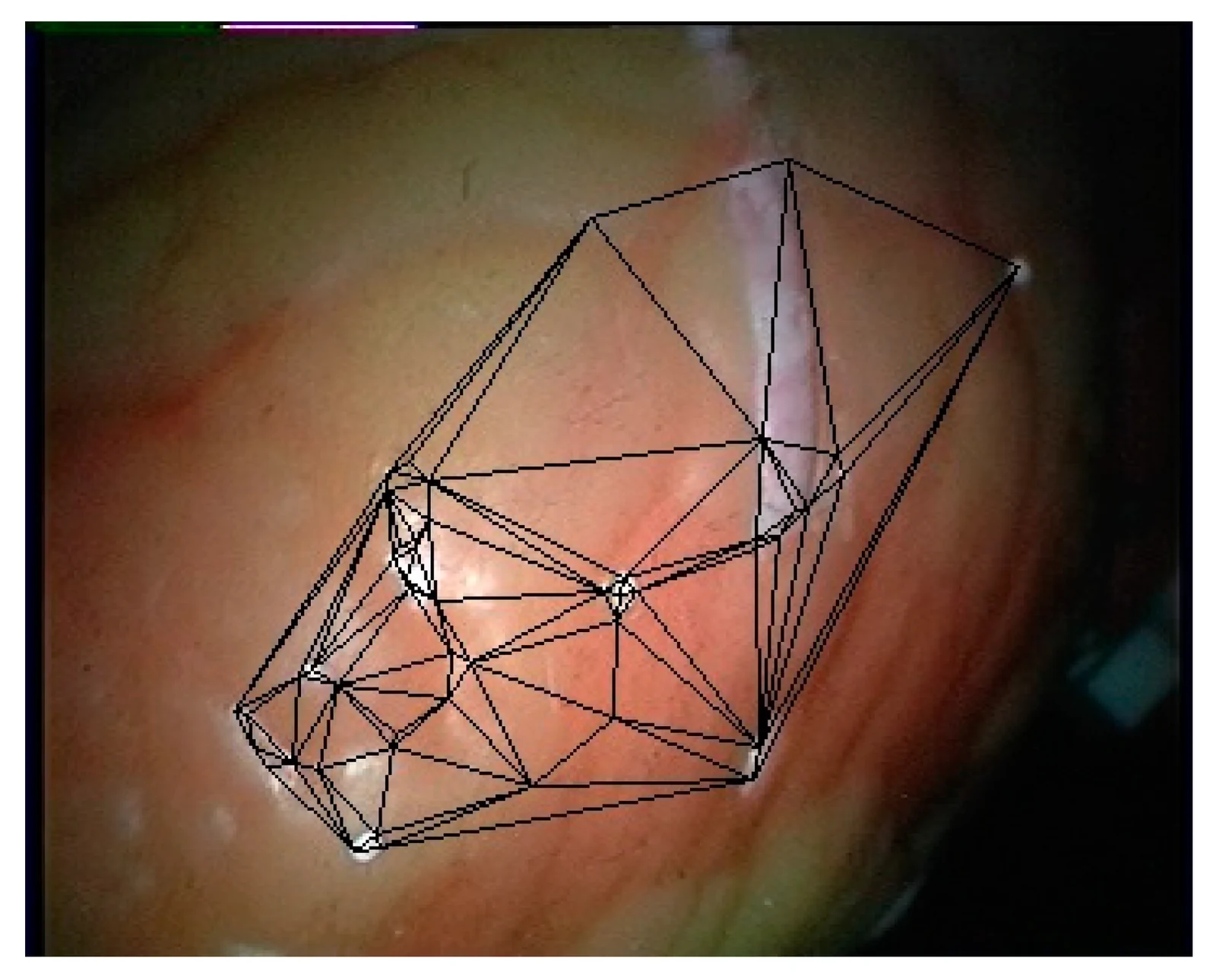
Triangulation results on the left image.

Firstly, the training data are generated from the first 200 frames of the left video of the endoscope to train the convolutional neural network. Then, the left and right images used for 3D curved surface reconstruction are matched with the model. Finally, the Delaunay triangulation of the feature points is performed using the plane projection method. To facilitate the calculation, the imaging plane of the image is taken as the projection plane, and the segmentation result on the left image is taken as the segmentation result of this frame. The triangulation result of the feature points in the left image on frame 201 is shown in Figure 11.

Because the endoscope image used in this paper is obtained using a parallel binocular camera, the 3D coordinates of all matching feature points can be calculated by using the geometric constraints of the parallel binocular camera. The plane triangulation relationship is mapped to the 3D discrete points, and then the texture mapping is carried out on each triangular surface. However, it can be seen from Figure 11 that the number of feature points obtained by this method is small, and its coverage area is also small. To obtain a more convenient visualization result, this paper smooths the joint of the triangle area, and extends the edge of the restoration surface to make the soft tissue surface display more widely.

**Figure 12:**
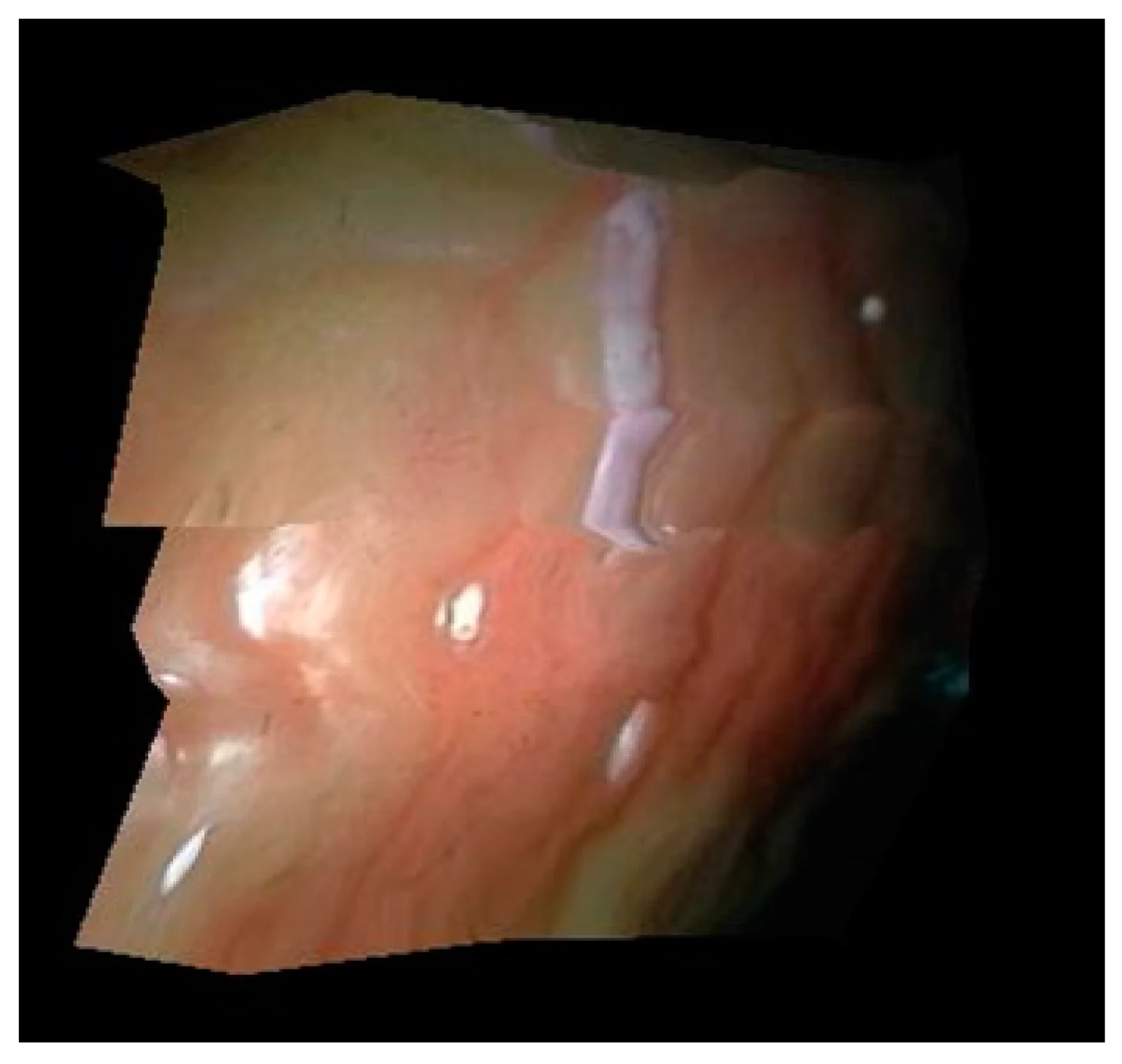
3D curved surface reconstruction result.

The 3D surface reconstruction results obtained by this method are shown in Figure 12. In this experiment, the focal length of the two cameras of the binocular endoscope is 1 mm, and the optical center distance of two cameras is 0.99 mm.

It can be seen from Figure 8 that the surface morphology of soft tissue can be recovered by this method. In this paper, multiple triangular regions are used to restore the tissue image, so the surface in the results will not be smooth. In addition, the surface texture connection dislocation exists in the restoration results, which is caused by less feature points collected by this method and a small amount of error in feature point extraction, but the surface reconstruction results roughly conform to the real situation of soft tissue surface.

### 3.2. Feature Point Tracking Experiment

**Figure 13:**
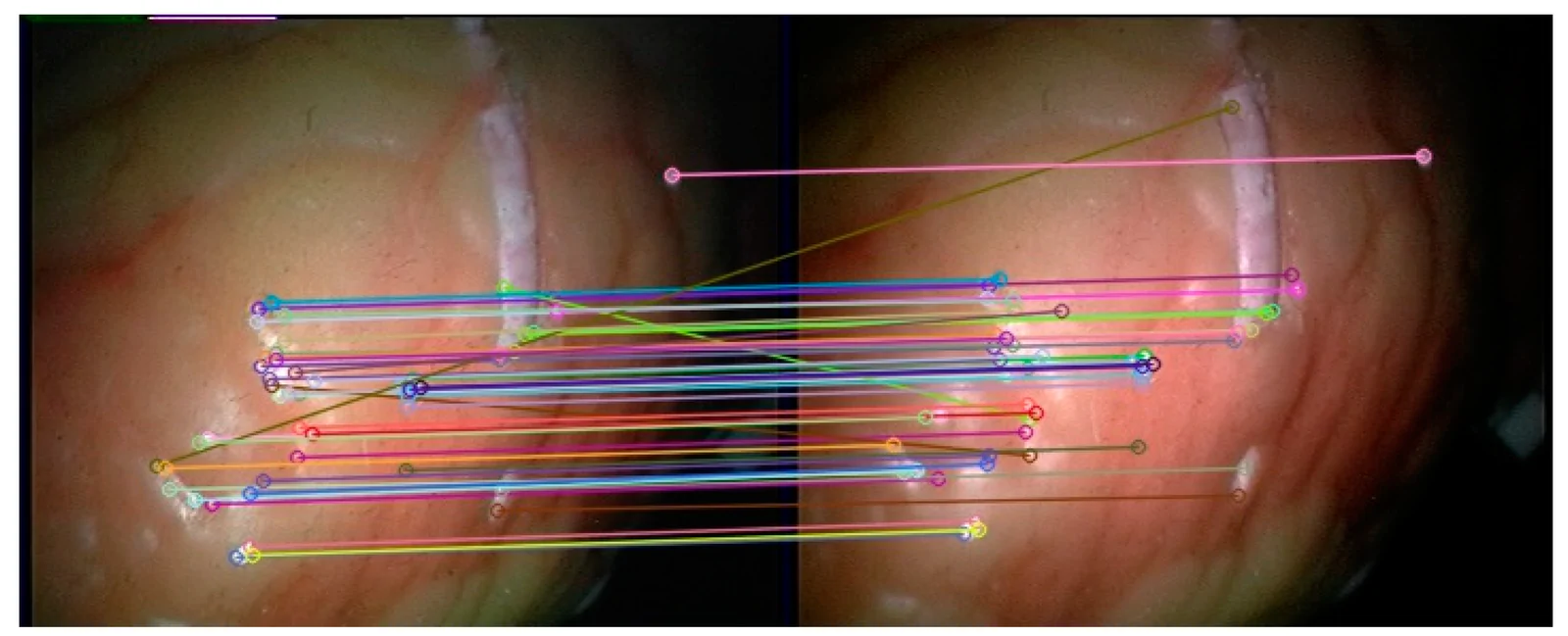
Feature matching results of the method in this paper.

The feature points in the left and right frames to be matched in the subsequent endoscopic video are extracted by the double decision tree FAST method based on C4.5 in this article, and then the feature points in each frame are classified through the trained convolutional neural network. In addition, feature matching is then performed. When the network feature selection threshold λ is set to 30%, 52 feature categories are obtained on the first frame of the endoscopic image. The matching effect on the left and right images in the 201st frame of the endoscopic video is shown in Figure 13.

It can be seen from Figure 13 that the feature matching method based on the convolutional neural network in this paper can effectively match the corresponding feature points in the left and right images of the subsequent frames, and the number of false matches is small, which proves the feasibility of the feature matching method in this paper. Since the method in this paper does not need to perform descriptor calculations on feature points when matching feature points, the SIFT, SURF and ORB feature detection algorithms with feature extraction and feature description are selected for comparison. These three methods all use brute force matching methods to perform feature matching. For the four methods, the left and right video sequences from the first frame to the 700th frame of the endoscopic video are selected for experiment, and the matching accuracy rate of the different methods and the average value of the matching time in each frame are obtained. Taking into account that the method in this paper uses the first 200 frames of the video to generate training data, the experimental results of the method in this paper will be counted on the first 200 frames and the last 500 frames. The experimental results are shown in Table 1.

Since there is no ground truth for any of the feature matching pairs in the experimental video, all the correct matching feature point pairs in the experimental results of this chapter are the results obtained after checking each matching result frame by frame. In Table 1, Method I and Method II in this article are the matching results of the method in the first 200 frames and the last 500 frames, respectively. It can be seen that the convolutional neural network used in this article is trained by using the first 200 frames of video to collect data. However, while the matching accuracy in the first 200 frames is higher than that in the subsequent 500 frames, time required to perform the matching is considerable.

It can be seen from Table 1 that in terms of the feature point pairs that are successfully matched, the number of matching pairs obtained by this method is relatively small compared with other methods. This is because the use of convolutional neural networks for feature classification limits the maximum number of matching point pairs when using this method. However, among the matched feature point pairs, there are very few false matches in the results of the method in this paper, and the matching accuracy rate is relatively high. In the results obtained by the ORB algorithm, the proportion of false matching pairs is the highest. In terms of computational time, the ORB algorithm with binary description is the most advantageous and takes the least time; the SURF algorithm is in the middle, while the SIFT algorithm requires the most time. Because the method in this paper needs to obtain the network output of each feature point in the feature matching process, and needs to further filter the results, the calculation time is between the SURF algorithm and the SIFT algorithm.

**Figure 14:**
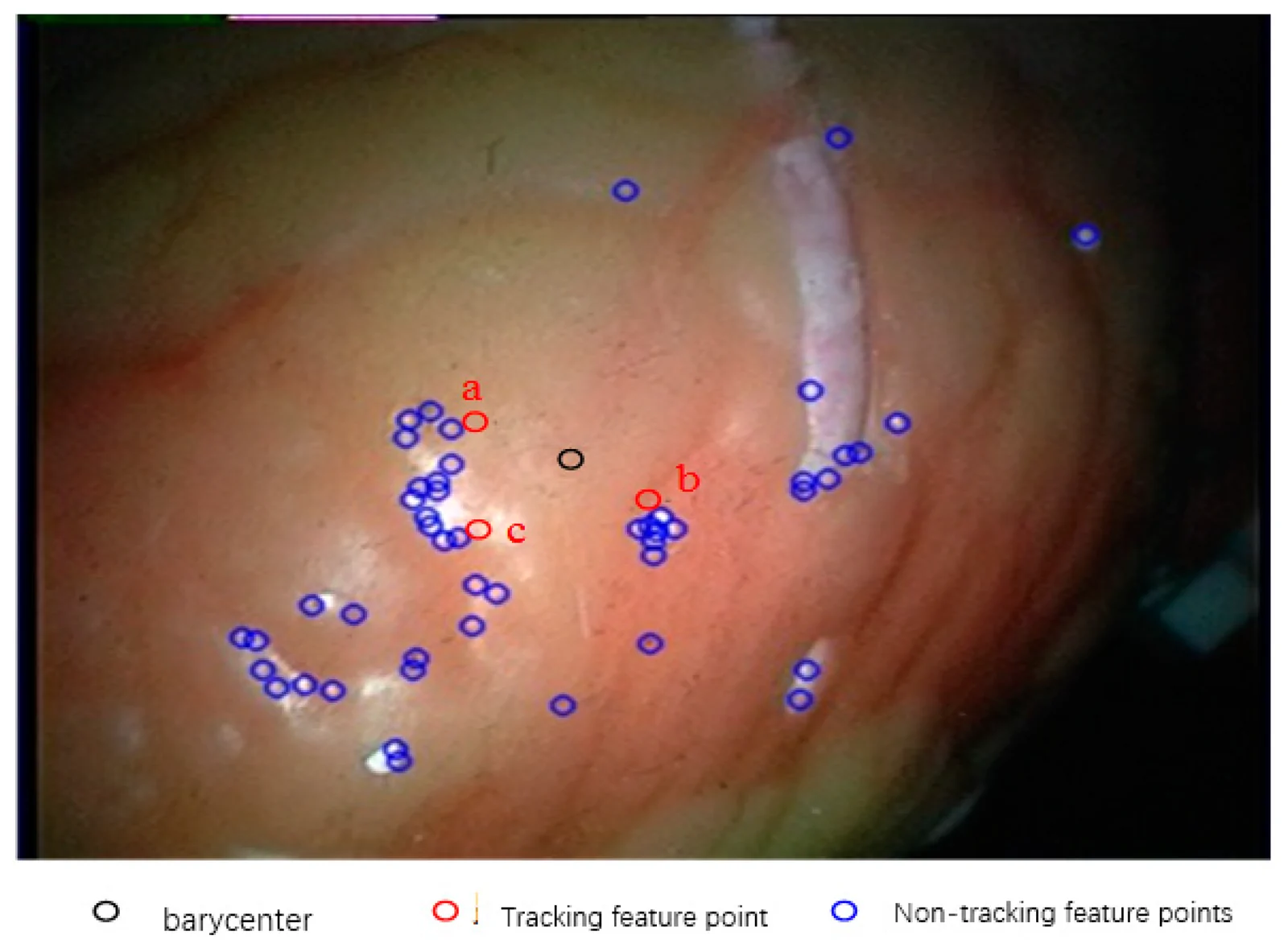
Selection of tracking points.

In this experiment, the number of feature points used in the initial frame of the endoscope video is 52. According to this method, the feature tracking experiment is carried out. Because of the large number of feature points tracked, and the fact that most of the feature points have similar motion, it is not necessary to display the tracking results of all feature points. In this paper, some of the feature points that are best able to represent the overall regional change are selected to display the results. Firstly, the centroids of all kinds of feature points on the starting frame are calculated, and then the three feature points closest to the centroid are selected as the tracking points of this experiment, and they are marked as a, b and c, as shown in Figure 14.

**Figure 15:**
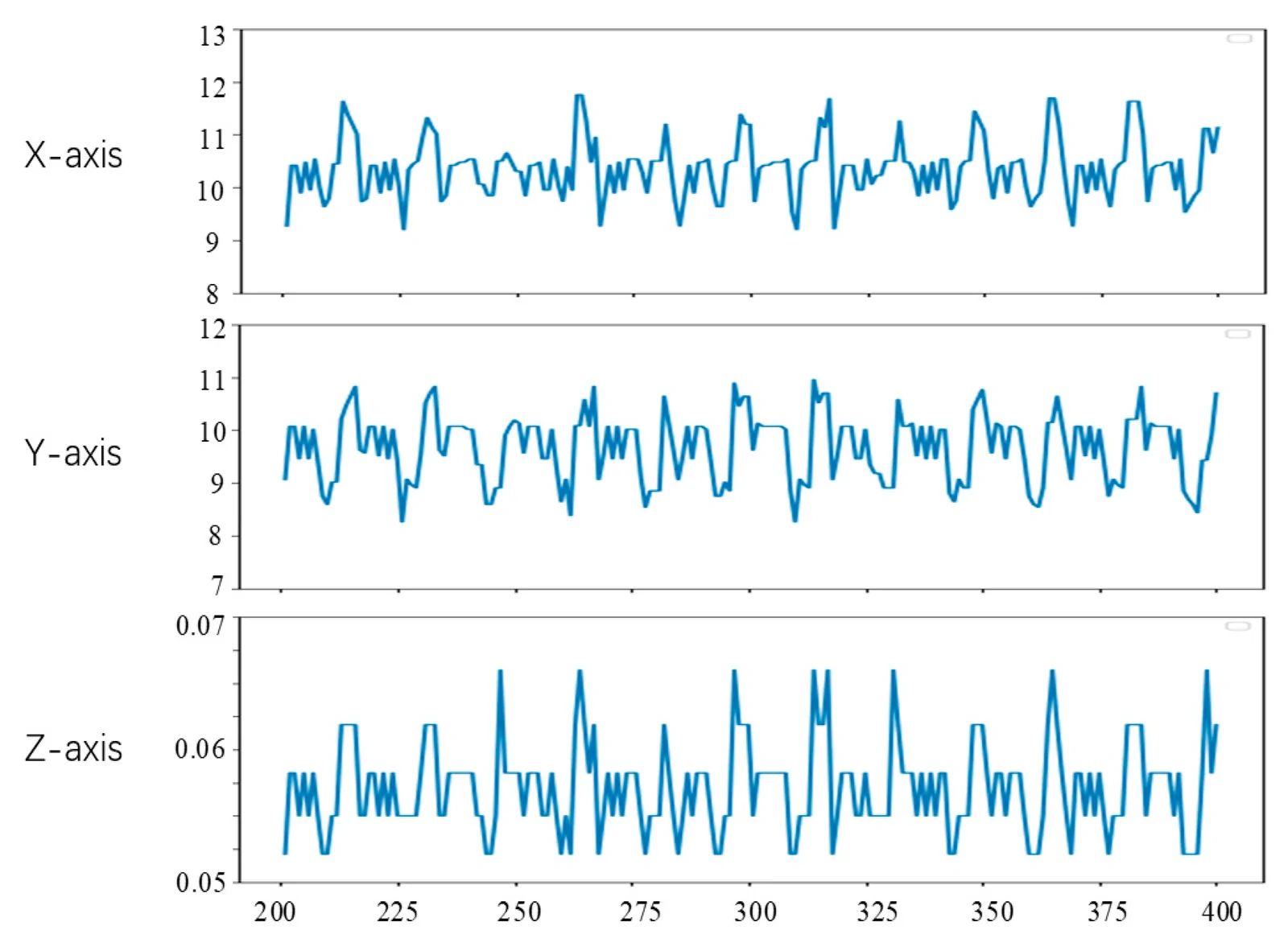
Point a motion tracking results.

**Figure 16:**
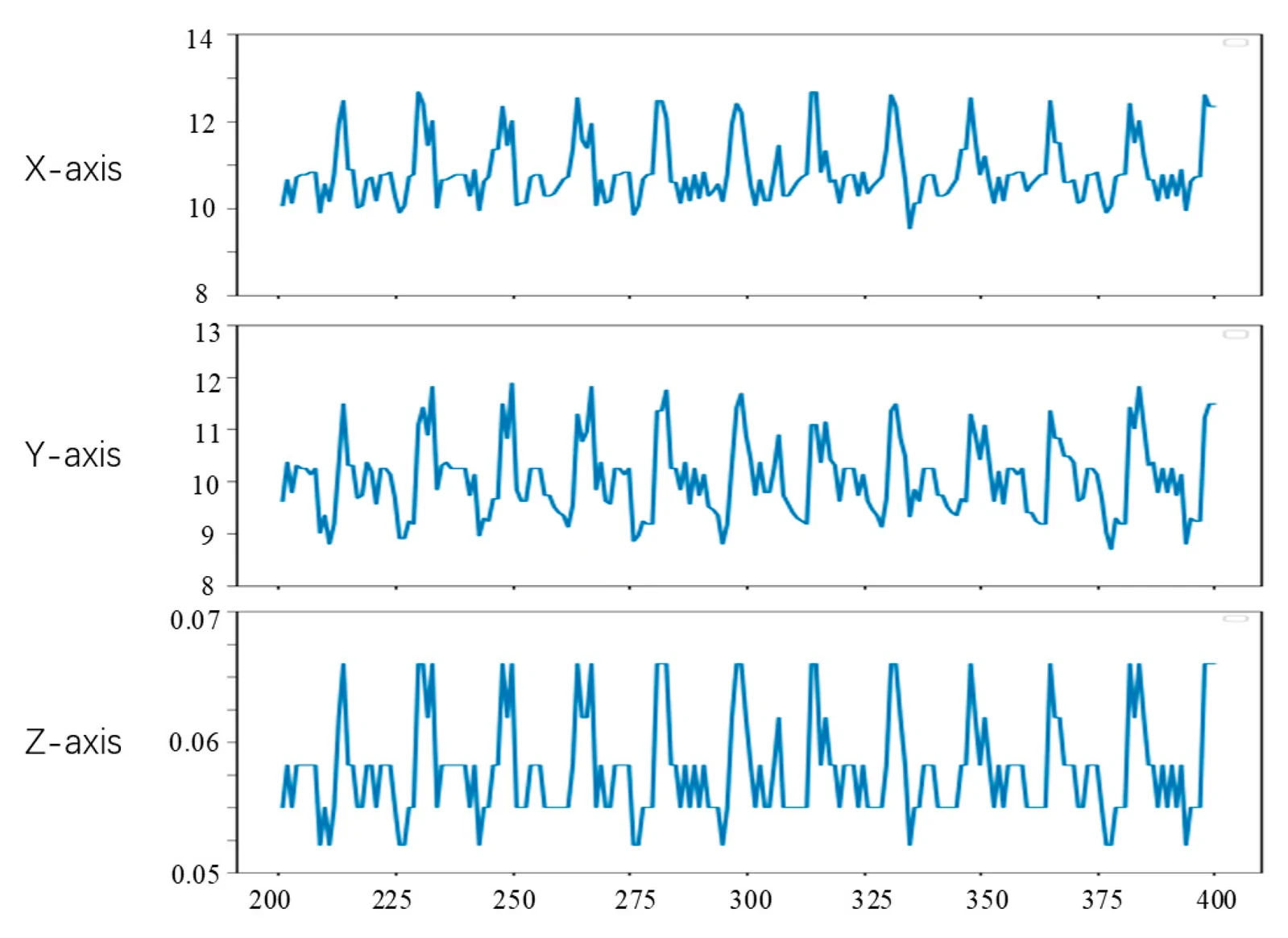
Point b motion tracking results.

**Figure 17:**
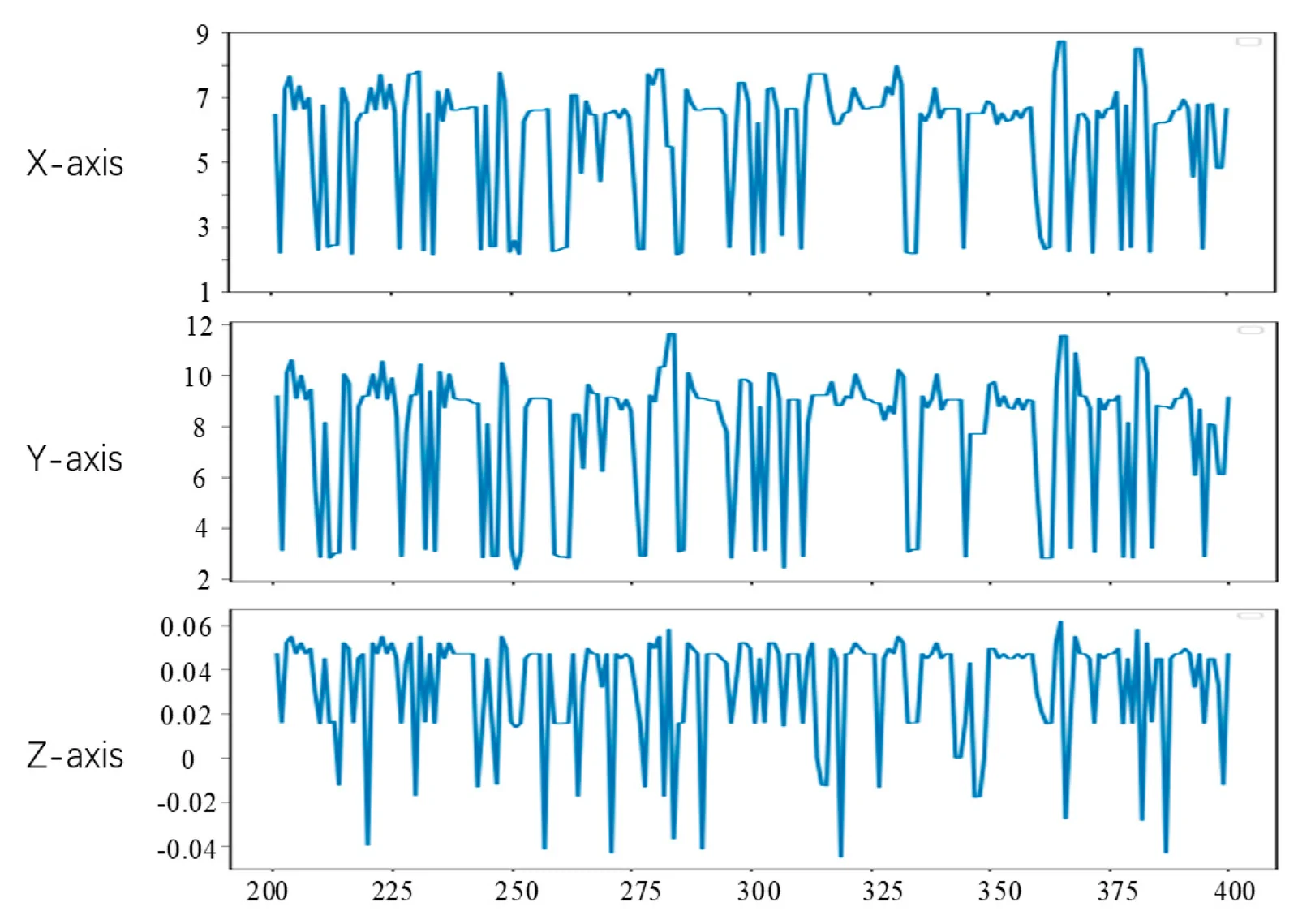
Point c motion tracking results.

Since the first 200 frames of the left video have been used to generate the training data of the convolutional neural network in the experiment, the feature tracking experiment in this section is carried out on the videos of frames 201 to 400. To observe the position changes of the feature points, we set the position of feature points in the lost tracking frame as the mean value of the position of the feature points in two adjacent frames. The tracking results of feature points a, b and c are shown in Figure 15, Figure 16 and Figure 17, respectively. The horizontal axis coordinate is the tracking frame number, and the vertical axis unit is millimeter.

## 4. Discussion

In this paper, a reconstruction method for points in three-dimensional space obtained using a binocular endoscope is studied. The three-dimensional coordinates of feature points are calculated by combining endoscope parameters and binocular space geometry. Delaunay triangulation is used to divide the discrete feature points into several triangles. The 3D surface reconstruction is realized by combining the 3D coordinates of the feature points. Then, feature tracking is realized by calculating the 3D coordinates of the same feature point in different frames using the feature matching method based on the convolutional neural network presented in this paper. Finally, 3D surface reconstruction and feature tracking are carried out on the endoscope image, and the results are analyzed, demonstrating the effectiveness of the proposed method.

In this experiment, the reconstruction of points in 3D space was achieved, and improvements demonstrated, but there are still some problems that require further research and optimization, for example: in this paper, the various parts of the reconstruction process were conducted separately, so each module interaction could also present some problems, and the performance evaluation was not performed in practical application. In future work, the overall performance and practical application of the algorithm will be studied further.

Besides, using pre-exiting surface reconstruction algorithms, several problems will emerge, for example, the surface holes, pits, humps, etc. For uneven point cloud data, more robust surface reconstruction algorithms are needed.

## 5. Conclusions

From Figure 15, Figure 16 and Figure 17, it can be seen that the method in this study is able to successfully track the position of the three selected feature points, and each feature point moves within a certain range, which shows the effectiveness of the feature tracking method presented in this paper. The tracking results of point a and point b show that both point a and point b move periodically. Due to the influence of feature point extraction algorithm, the tracking results contain a small amount of noise. However, the tracking results of point c show that the movement of this point does not show obvious regularity in the current stage.

From Figure 15 and Figure 16, it can be seen that the two points a and b had completed about 12 periodic movements at 400 frames. In combination with the frame rate of 25 frames per second of endoscope video, i.e., about 80 beats per minute, which is consistent with the beating of a simulated silica gel heart. Furthermore, because the distance between point a and point b on the soft tissue surface is relatively close, the motion amplitude of the two points is also very similar. The ranges of motion of a and b on the X, Y and Z axes are about 3 mm, 3 mm and 0.02 mm, respectively, which is also roughly consistent with the surface motion state of the heart.

It can be seen from Figure 17 that the tracking results of point c do not show obvious periodicity, which is due to the existence of a few irregular motion areas on the surface of the tracked soft tissue. The motion ranges of point c on the X, Y and Z axes are 6 mm, 10 mm and 0.1 mm, respectively. Compared with the motion ranges of points a and b, the movement of c is more intense, but its coordinates still change within a certain range.

Through the tracking results of points a, b and c, we can know that there are some feature points with similar motion on the surface of the heart, but some feature points move irregularly, and the motion amplitude of different feature points is not the same. In the actual operation video, there will be more accidental factors, and feature point tracking is more difficult.

## Figures and Tables

**Table 1:** Feature matching results of various methods.

Method	SIFT	SURF	ORB	Method I	Method II
Successfully matched feature point pairs	44	118	233	50	49
Match the feature point pairs correctly	37	92	168	45	43
Matching accuracy rate (%)	84.09	77.97	72.10	90.00	87.76
Total matching time (ms)	93.31	46.59	27.26	76.03	76.01

## Data Availability

The data used in this paper is an open-source data provided by the Hamlyn Center at Imperial College London at hamlyn.doc.ic.ac.uk/vision (accessed on 9 November 2021).
