# Correction: Zhang et al. Study on Reconstruction and Feature Tracking of Silicone Heart 3D Surface. *Sensors* 2021, *21*, 7570

**DOI:** 10.3390/s26154753

**Published:** 2026-07-27

**Authors:** Ziyan Zhang, Yan Liu, Jiawei Tian, Shan Liu, Bo Yang, Longhai Xiang, Lirong Yin, Wenfeng Zheng

**Affiliations:** 1 School of Innovation and Entrepreneurship, Xi’an Fanyi University, Xi’an 710105, China; ziyaan.zhang@outlook.com; 2 School of Automation, University of Electronic Science and Technology of China, Chengdu 610054, China; eevian.liu@gmail.com (Y.L.); jravis.tian23@gmail.com (J.T.); yangbo.sd@gmail.com (B.Y.); longhai.xiang@outlook.com (L.X.); wenfeng.zheng.cn@gmail.com (W.Z.); 3 Department of Geography and Anthropology, Louisiana State University, Baton Rouge, LA 70803, USA; yin.lyra@gmail.com

## References Correction

In the original publication [1], References 5–17, 22–23, 25, and 31–34 were identified as unrelated or inappropriate to the study. These citations have been removed.

To provide a more accurate background, seven authoritative review articles have been added and are now listed as References [5–11] in the revised manuscript.

5. Lim, S.P.; Haron, H. Surface reconstruction techniques: A review. *Artif. Intell. Rev.* **2014**, *42*, 59–78. https://doi.org/10.1007/s10462-012-9329-z.
6. Guo, X.; Xiao, J.; Wang, Y. A survey on algorithms of hole filling in 3D surface reconstruction. *Vis. Comput.* **2018**, *34*, 93–103. https://doi.org/10.1007/s00371-016-1316-y.
7. Paoli, A.; Neri, P.; Razionale, A.V.; Tamburrino, F.; Barone, S. Sensor Architectures and Technologies for Upper Limb 3D Surface Reconstruction: A Review. *Sensors* **2020**, *20*, 6584. https://doi.org/10.3390/s20226584.
8. Berger, M.; Tagliasacchi, A.; Seversky, L.M.; Alliez, P.; Guennebaud, G.; Levine, J.A.; Sharf, A.; Silva, C.T. A Survey of Surface Reconstruction from Point Clouds. *Comput. Graph. Forum* **2017**, *36*, 301–329. https://doi.org/10.1111/cgf.12802.
9. Han, X.F.; Laga, H.; Bennamoun, M. Image-Based 3D Object Reconstruction: State-of-the-Art and Trends in the Deep Learning Era. *IEEE Trans. Pattern Anal. Mach. Intell.* **2021**, *43*, 1578–1604. https://doi.org/10.1109/TPAMI.2019.2954885.
10. Zhou, H.; Jagadeesan, J. Real-Time Dense Reconstruction of Tissue Surface from Stereo Optical Video. *IEEE Trans. Med. Imaging* **2020**, *39*, 400–412. https://doi.org/10.1109/TMI.2019.2927436.
11. Chen, L.; Tang, W.; John, N.W.; Wan, T.R.; Zhang, J.J. SLAM-based dense surface reconstruction in monocular Minimally Invasive Surgery and its application to Augmented Reality. *Comput. Methods Programs Biomed.* **2018**, *158*, 135–146. https://doi.org/10.1016/j.cmpb.2018.02.006.

With this correction, the order of some references has been adjusted accordingly. The authors state that the scientific conclusions are unaffected. This correction was approved by the Academic Editor. The original publication has also been updated.
